# Effect of music at 432 Hz and 440 Hz on dental anxiety and salivary cortisol levels in patients undergoing tooth extraction: a randomized clinical trial

**DOI:** 10.1590/1678-7757-2019-0601

**Published:** 2020-05-11

**Authors:** Pedro Christian ARAVENA, Camila ALMONACID, Marcelo Ignacio MANCILLA

**Affiliations:** 1 Instituto de Anatomía, Histología y Patología Facultad de Medicina Universidad Austral de Chile Valdivia Chile Instituto de Anatomía, Histología y Patología . Facultad de Medicina . Universidad Austral de Chile . Valdivia , Chile .; 2 Escuela de Odontología Facultad de Medicina Universidad Austral de Chile Valdivia Chile Escuela de Odontología . Facultad de Medicina . Universidad Austral de Chile . Valdivia , Chile .; 3 CPO São Leopoldo Mandic Departamento de Implantologia CampinasSão Paulo Brasil CPO São Leopoldo Mandic , Departamento de Implantologia , Campinas , São Paulo , Brasil .; 4 Universidad de La Frontera Facultad de Odontología Programa de Especialización en Trastornos Temporomandibulares y Dolor Orofacial Temuco Chile Universidad de La Frontera , Facultad de Odontología , Programa de Especialización en Trastornos Temporomandibulares y Dolor Orofacial , Temuco , Chile .

**Keywords:** Music therapy, Dental anxiety, Hydrocortisone, Oral surgery, Clinical trial

## Abstract

**Objective:**

The aim of this study was to compare the effects of music at 432 Hz, 440 Hz, and no music on the clinical perception of anxiety and salivary cortisol levels in patients undergoing tooth extraction.

**Methodology:**

A parallel-group randomized clinical trial was conducted. Forty-two patients (average age: 23.8±7.8 years, 27 women) with a moderate level of anxiety were distributed in three groups: use of music for 15 minutes at a frequency of 432 Hz (n=15), at 440 Hz (n=15) and a control group without music (n=12). The CORAH Dental Anxiety Scale and salivary cortisol levels, estimated by the solid phase enzyme-linked immunosorbent assay (ELISA), were measured and compared before and after the music intervention between groups (two-way ANOVA-Tukey p<0.05, RStudio).

**Results:**

Significantly lower anxiety level values were observed at 432 Hz (8.7±2.67) and 440 Hz (8.4±2.84) compared to the control group (17.2±4.60; p<0.05). The salivary cortisol level at 432 Hz (0.49±0.37 μg/dL) was significantly lower than 440 Hz (1.35±0.69 μg/dL) and the control group (1.59±0.7 μg/dL; p<0.05).

**Conclusion:**

The use of music significantly decreased clinical anxiety levels, and the frequency of 432 Hz was effective in decreasing salivary cortisol levels before tooth extraction.

## Introduction

Dental care is considered one of the five most commonly feared situations and one of the main reasons for missing dental appointments. ^1 , 2^ Music therapy has been used as a non-pharmacological method to control anxiety ^3 , 4^ due to its suppressive action on the sympathetic nervous system, leading to a reduction in both adrenergic activity and neuromuscular activation, ^5 , 6^ thus reducing the patient’s anxiety. ^6 , 7^

In dentistry, the use of music has proven to reduce the physiological parameters of anxiety in patients during dental cleaning, ^8^ extractions, ^9 , 10^ endodontic treatments, ^11^ and pediatric care. ^12^ One of the most objective and simple ways to measure stress and anxiety is through salivary cortisol. ^13^ Studies have shown that music significantly reduces saliva cortisol levels during simulated dental care situations, such as showing the patient the carpule syringe needle and exposure to a high-speed dental handpiece sound. ^10^ In this regard, it has been described that music is effective in controlling anxiety. ^11 , 14^ According to the International Organization for Standardization (ISO) ^15^ , the pitch standard established for the musical note A is 440 Hz. However, it has been noted that the tones of the 440 Hz tuning frequency can be uncomfortable, irritating and disagreeable, whereas the intervals and tones obtained from the 432 Hz tuning frequency are peaceful, pleasant and more harmonious, ^16^ suggesting that the A note should be optimally tuned to 432 Hz because it exerts less pressure on the singers’ voices and has greater musical qualities. ^17^

To date, there have been no reports comparing the effect of a type of music with different frequencies to control anxiety in patients during dental treatment. Considering that the measurement of salivary cortisol allows to identify the stress levels of patients undergoing dental extractions, ^18 , 19^ this study aimed to evaluate the effect of music at 432 Hz and 440 Hz, versus no music, on anxiety levels according to the CORAH Modified Dental Anxiety Scale (CORAH-MDAS), ^20 , 21^ and the salivary cortisol levels of patients requiring a simple dental extraction.

## Methodology

### Study design

A parallel-group randomized clinical trial was conducted. The study protocol was reviewed and approved by the Scientific Ethics Committee of the Health Service in Valdivia, Chile (Ref nº 195/2018). The methodology was performed within the parameters of the CONSORT ^22^ statement guidelines and registered for clinical trials in the ISRCTN registry (nº ISRCTN28195632).

### Subjects and sample size

We included healthy patients who were ASA I, between 15 and 40 years of age, with indication of a simple dental extraction, treated in the oral surgery section of the Dental Clinic of the Universidad Austral de Chile between June and September 2018 in Valdivia, Chile. Patients with systemic diseases that can directly affect the physiological variables associated with anxiety were excluded: diabetes, immunodepression, high blood pressure, thyroid pathology, cardiac diseases, alcoholism, heavy smoker (more than 10 cigarettes per day), those receiving permanent pharmacological treatment with tricyclic antidepressants, anticholinergics, benzodiazepines, antihypertensives, synthetic glucocorticoids (prednisone and prednisolone) or anticonvulsants; as well as pregnant women and patients with hearing impairment. Patients who had pericoronitis or acute infection of the tooth to be extracted at the time of the surgery or ten days prior were also excluded.

The sample size was calculated based on the results of the effect of music on salivary cortisol conducted by Mejía-Rubalcava, et al. ^10^ (2015). An alpha error of 5%, a beta error of 80% and a mean difference of 0.8±0.9 µg/dL were considered for study groups. According to these data, each group determined a minimum number of 12 subjects (algorithm “power one mean 1.3 0.5, sd(0.9)” Stata/MP v14.0. STATACorp. TX. USA)

### Study intervention

Patients who met the inclusion criteria were invited to voluntarily participate in the study and they accepted by signing an informed consent. To define a baseline state of anxiety, a researcher (CA) selected patients who presented a moderate or high score of anxiety at the CORAH-MDAS ^20 , 21^ applied at the time of invitation. To evaluate, with local participants, the transcultural language change of the original scale, a facial validity analysis was performed on ten patients at the University’s dental clinic, directly consulting on the understanding and format of the instrument. All of them stated that they understood the text, not suggesting changes.

Another blinded researcher (PCA) in charge of the statistical data analysis performed simple random sampling, according to the sequence of numbers randomized by computer that was assigned to each patient upon entering the dental extraction service, to determine which group the patient belonged to, using the “RANDBETWEEN” function in Microsoft Excel ^™^ v.2016 (Microsoft Corp., Redmond, WA, USA). The first group was subjected to musical stimulation at 432 Hz, the second group at 440 Hz, and the third was a control group with no musical intervention. This created double blinding of both the investigator in charge of the results analysis and the study participants.

### Salivary cortisol and anxiety measurement

All the interventions and measurements were performed between 2 and 4 p.m. to monitor cortisol levels according to the circadian cycle. ^19^ Patients answered the CORAH-MDAS questionnaire, and a saliva sample was taken with the Salivary Cortisol Immunoassay Kit (Salivary Cortisol ELISA kit, Salimetrics Assays ^™^ , Pennsylvania, USA) and placed in a previously sterilized polyethylene container. Based on randomization and previous recommendations on the use of music in dental surgeries, ^9^ each patient received Beats ^™^ Solo 3 Wireless headphones (Copyright ^™^ 2017 Apple Inc. All rights reserved) with active noise control connected to a Bluetooth-enabled audio player. Participants were exposed for 15 minutes to music played at 432 Hz or 440 Hz depending on the assigned group, using for this purpose two songs by Giorgio Costantini - 2012 from the album “Universound: 432 Hz” (retrieved in the 432 Hz ^23^ and 440 Hz ^24^ versions).The same protocol was used in the control group but without music in their headphones. The earphones were then removed, and the patient was asked to respond the CORAH-MDAS questionnaire a second time and a second saliva sample was taken. The samples were stored at -20°C for later analysis in the Immunology Laboratory of the Universidad Austral de Chile Medical School.

### Salivary cortisol analysis

The samples were defrosted and centrifuged at 3000 rpm for 5 minutes. The cortisol concentration of the samples was quantified using an immunoassay kit, and validated following the instructions provided by the manufacturer. A 2 mL sample of supernatant was obtained and placed in a sterile 10 mL polyethylene container. The optical density was read on a microplate reader at 450 nm (ELx800; BioTeck ^™^ Instruments Inc., Vermont, USA). All the samples were analyzed in a single step and in duplicate.

### Statistical analysis

To evaluate the effectiveness of musical stimulation on anxiety and salivary cortisol levels, the three study groups were considered an independent variable. Dependent variables were the CORAH-MDAS anxiety scale and the salivary cortisol levels measured in µg/dL. A Shapiro-Wilk test was used to verify data normality. To verify the difference between the study groups before and after the clinical trial, a two-way ANOVA, *post-hoc* Tukey test was used. Covariance analysis and a linear regression model (ANCOVA; p<0.05) were used to verify the effect size of the initial anxiety and salivary cortisol values on the effect of the music on the final measurements. Data were tabulated and analyzed using R (R Core Team) with tidyverse ^25^ and nlme ^26^ packages.

## Results

**Figure 1:**
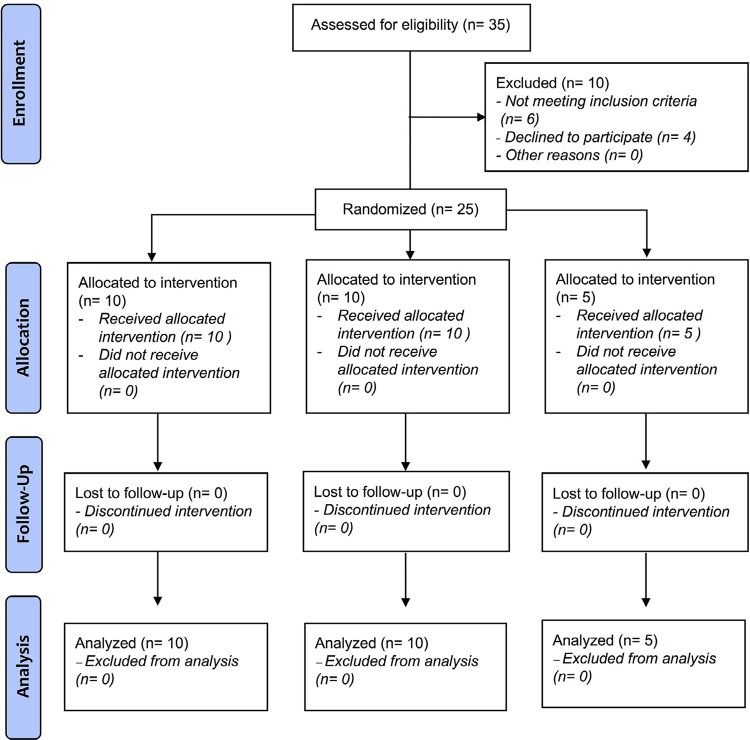
Flow chart of phases of parallel-group randomized clinical trial

Forty-two patients were included in the study with 23.8±7.8 as the average age (range 15 – 40 years; 64.2% women) randomized in three groups: 432 Hz (n=15), 440 Hz (n=15), Control (n=12). No significant differences in sex or age were found between the groups ( Table 1 , Figure 1 ).

**Table 1:** Age and sex according to study group

	Study group			
Variable	432 Hz (n=15)	440 Hz (n=15)	Control (n=12)	p value
mean age±SD (in years)	23.2±6.37	23.2±6.37	23.8±8.98	0.11 ^a^
Female sex (%)	9 (60)	10 (66.6)	8 (66.6)	0.28 ^b^

SD: Standard Deviation; a. One-way ANOVA (p<0.05); b. Chi-squared test (p<0.05)

**Figure 2:**
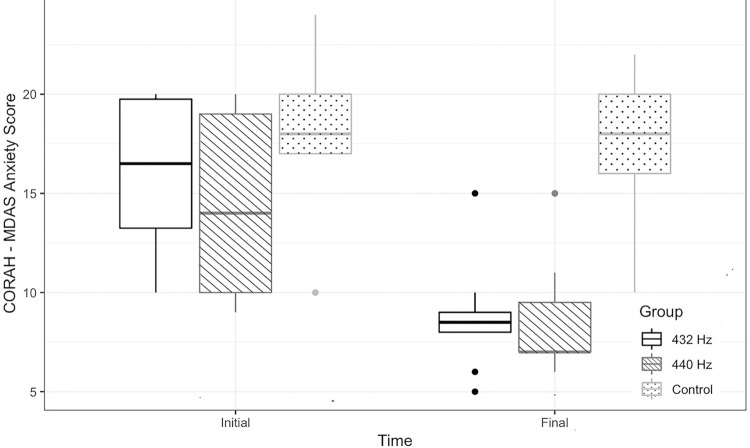
Score of the Modified CORAH Dental Anxiety Scale by group over time (Two-way ANOVA; *statistical significance p<0.05)

**Figure 3:**
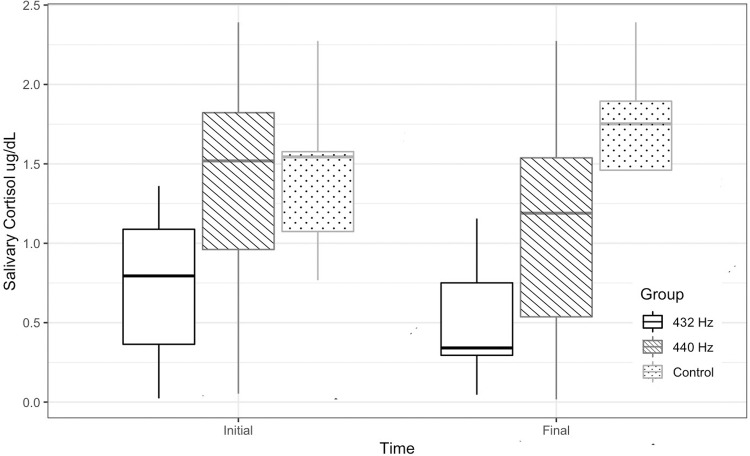
Salivary cortisol measurement by group over time (Two-way ANOVA; *statistical significance p<0.05) *statistical significance p<0.05)

A significant reduction in anxiety levels was observed according to the CORAH-MDAS in the 432 Hz and 440 Hz groups (p<0.0001) considering the baseline indices prior musical intervention. However, no significant changes were observed in the salivary cortisol levels with music at 432 Hz, 440 Hz, or the Control group ( Table 2 ) ( Figures 2 and 3 ).

**Table 2:** Comparison of variables by groups at the beginning and end according to two-way ANOVA

Variable		Group		
		432 Hz (n=15)	440 Hz (n=15)	Control (n=12)
CORAH-MDAS Anxiety	Initial	16.1±3.75	14.3±4.55	17.8±5.12
	Final	8.7±2.67	8.4±2.84	17.2±4.60
	Mean difference	7.45	5.91	0.61
	p-value	0.001*	0.014*	0,99
Salivary Cortisol (µg/dL)	Initial	0.74±0.45	1.35±0.69	1.44±0.57
	Final	0.49±0.37	1.06±0.72	1.59±0.70
	Difference	0,249	0,289	-0,151
	p-value	0,93	0,87	0,99

*Statistically significant differences as measured by two-way ANOVA (p<0.05)

The linear regression model showed that the initial anxiety (F=41.36; p<0.001) and salivary cortisol (F=40.87; p<0.001) levels were significantly lower for the final values observed. The effect size of the participants’ initial anxiety levels was 0.58 times (95% CI: 0.32-0.78; p<0.001) in the observed result, mainly in the control group (coefficient: 7.11; 95% CI: 4.8-9.42; p<0.001). The effect size of the initial values of salivary cortisol was 0.9 times (95% CI: 0.7-1.1; p<0.001) in the observed result, mainly in the control group (coefficient: 0.45; 95% CI: 0.12-0.79; p: 0.01). The detail of the effect sizes and their statistical significance are in Table 3 .

**Table 3:** Linear regression and co-variable analysis of the patient variables fit to initial anxiety levels, initial cortisol levels and study group

Variable	Co-Variable	Coefficient (standard error)	95% CI	p-value
Final Anxiety	Initial Anxiety	0.58 (0.96)	0.38 - 0.78	< 0.001*
432 Hz (base)	440 Hz	-0.04 (0.89)	-3,71	0,95
	Control group	7.11 (1.11)	4.8 - 9.42	<0.001*
Final Cortisol	Initial Cortisol	0.91 (0.09)	0.70 - 1.11	< 0.001*
432 Hz (base)	440 Hz	0.0009 ( 1.3)	-0,54	0,99
	Control	0.45 (0.16)	0.12 - 0.79	0.01*

*Statistically significant differences as measured by linear regression model (p<0.05)

## Discussion

This study analyzed the use of music at 432 Hz and 440 Hz to control anxiety during dental care procedure. Results showed that the clinical perception of anxiety of the participants in the music intervention groups was significantly lower than the control group; and that the subjects exposed to music at 432 Hz presented significantly lower salivary cortisol levels than the control group (p<0.05).

Anxiety caused by dental treatment is a complex issue conditioned by personality characteristics, fear of pain, past traumatic dental experiences particularly in childhood, or the influence of anxious relatives, ^1 , 2^ which manifests as increased anxiety and salivary cortisol levels before tooth extractions. ^19 , 20^ However, music has proved to be effective in anxiety control. ^8 - 10 , 12^ Di Nasso, et al. ^11^ (2016) studied the impact of music at 432 Hz in patients undergoing endodontic treatment, showing that blood pressure and heart rate decreased significantly compared to a control group, suggesting that music affects the autonomic nervous system, and the patient’s musical preference could have a greater positive impact than the operator’s.

The explanation for the effect of music tuned at 432 Hz for anxiety control could rely on the spectral centroid theory, ^27^ which postulates that the musical note A=432 Hz contains different or superior sound qualities, and details how the perception of a sound can be drastically modified when the frequency spectrum changes. Our results showed that music at both 432 Hz and 440 Hz significantly reduced clinical anxiety levels according to the CORAH-MDAS compared to the control group; and that emotional response and perception of anxiety when using the CORAH-MDAS do not show significant differences between musical frequencies (432 Hz – 440 Hz). Therefore, the reduction in clinical stress awareness is probably linked to the listener’s interpretations, his or her associations, and mental constructions, rather than to any innate pitch attribute. However, regarding the physiological parameter of salivary cortisol, we observed that music at 432 Hz had significantly smaller concentrations than the control group ( Figure 3 ). We found similar results on plasma cortisol levels during and three postoperative hours in patients undergoing general surgery, ^28^ suggesting a physiological explanation because nitric oxide may be responsible for reducing anxiety and stress in response to music therapy, probably as part of a complex interrelationship between emotional centers within the central nervous system. ^29^ On the other hand, a report by Stefano, et al. ^30^ (2004) showed that there was a statistically significant increase in mu opiate receptors in mononuclear cells in subjects undergoing pre and post-musical intervention, finding that IL-6 levels were significantly lower in peripheral blood plasma samples compared to the control group.

The authors define the type of music applied in musical medicine studies as “relaxing”, such as classical music or music described as “calming for the patient” ^10^ , the melodies of pianos and guitars, ^11^ latin chant and non-musical acoustic control (e.g., sound of waves), ^14^ which allow a standardization in the effect of anxiety control. A meta-analysis by Pelletier ^31^ (2004) concludes that musical stimuli selected arbitrarily have a more significant effect on stress reduction than music chosen by the patients themselves, because they might be associated with an event that induces a previously conceived emotion. Therefore, it stimulates the patient rather than increase relaxation. Moreover, according to the effect of music on physiological responses a study by Bradt, et al. ^4^ (2013) suggests that listening to music has a small impact on heart rate variability, blood pressure and respiratory frequency, but more research on this topic is needed. As such, the medical professional should adapt the musical therapy to the present needs of the patients to provide an experience of support, inclusion and acceptance. ^4^

This study’s limitations are related to the lack of a previous protocol that clearly indicates the type of music, the time and the suitable methods of use to conduct clinical trials on the effect of music on anxiety control. Also, the possible risk of bias in variables inherent to the patient, such as personal taste in music, current psychological state or previous anxiety experiences during dental treatment may cause changes in the results, given the subjectivity in the music selection. So, it is necessary to standardize these criteria. On the other hand, comparing music intervention at different frequencies is somewhat controversial, mainly because no other study provides a precedent that different frequencies of the same musical composition are more pleasant or harmonious. Despite these limitations, our study shows the levels of anxiety and salivary cortisol before and after musical intervention at different frequencies, being the first report in our area to determine the effectiveness of music therapy at varying frequencies to control dental treatment anxiety.

## Conclusion

Musical intervention at 432 Hz during dental treatment proved to reduce salivary cortisol levels in comparison to its absence, without showing changes in the clinical perception of anxiety in patients. Thus, medicine musical therapy can be considered a non-invasive, economic, and effective intervention to reduce anxiety levels in patients before a dental procedure. Future studies should compare other musical frequencies, as well as the duration of music therapy, type of dental intervention and the use of different physiologically sensitivities, such as verbal information (warning) before dental procedures. ^32^
